# General anaesthetics induce tonic inhibition and modulate the gain of neural populations : a modeling study

**DOI:** 10.1186/1471-2202-13-S1-P16

**Published:** 2012-07-16

**Authors:** Axel Hutt, Thomas Voegtlin

**Affiliations:** 1 INRIA CR Nancy - Grand Est, CS20101, 54603 Villers-ls-Nancy Cedex, France

## 

Anaesthetic agents are known to affect extra-synaptic GABAergic receptors[1], which induce tonic inhibitory currents. Since these receptors are very sensitive to small concentrations of agents, they are supposed to play an important role in the underlying neural mechanism of general anaesthesia. Moreover anaesthetic agents modulate the encephalographic activity (EEG) of patients and hence show an effect on neural populations. To understand better the tonic inhibition effect in single neurons on neural populations modulating the EEG, the work considers a neural population in a steady-state and studies numerically and analytically the modulation of its population firing rate and the nonlinear gain with respect to different levels of tonic inhibition. We consider populations of both type-I and type-II neurons. The populations under study are heterogeneous involving distributions of firing thresholds and inhibitory conductances. The tonic inhibition introduces shunting action.

The work reveals an increase of the population gain by increasing tonic inhibition and discovers a maximum of the gain subjected to the level of inhibitory heterogeneity, cf. Fig.1 for a population of type-I neurons. Here we have implemented Integrate-and-Fire models including constant excitatory, inhibitory and leaky conductances. In addition to the numerical study, we derive analytical expressions for the population firing rate in the presence of the inhibitory heterogeneities. All numerical results show good accordance to the corresponding analytical expressions. The modeling results obtained show that tonic inhibition increases the firing threshold and augments the responsivenss of neural populations to external stimuli.

**Figure 1:**
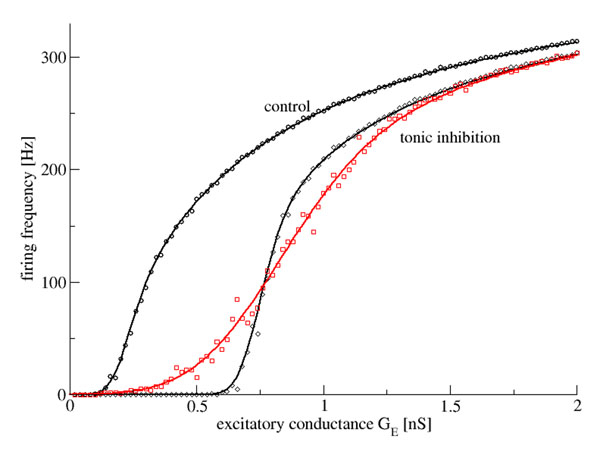
The population firing rate in a population of type-I neurons plotted with respect to the conductance at excitatory synapses *G_E._* The tonic inhibition shunts the membrane at extra-synaptic inhibitory receptors in average by 0.1nS. The heterogeneity of the shunting conductances have standard deviations 0.1nS (black) and 0.5nS (red).

Summarizing, the present work has discovered an increase of the population firing threshold and a population gain modulation induced by tonic inhibition and presents a mathematical expression for the population firing rate. Since extra-synaptic receptors nduce tonic inhibition and are activated by several general anaesthetics, it is reasonable to reason that neural populations under anaesthetic action experience both effects. Consequently, we conclude that anaesthetics may both diminish the resting activity by shift of the firing threshold [2] and disrupts functionally neural circuits by increased population gain [3].

## References

[B1] Orser B Extrasynaptic GABAA receptors are critical targets for sedative-hypnotic drugs J. Clin. Sleep Med 2006 2 S12 8 17557502

[B2] Steyn-Ross M Steyn-Ross D Sleigh J Modelling general anaesthesia as a first-order phase transition in the cortex Prog. Biophys. Molecul. Biol 2004 85 2- 3 369 385 10.1016/j.pbiomolbio.2004.02.001 15142753

[B3] Le Masson G Renaud-Le Masson S Debay D Bal T Feedback inhibition controls spike transfer in hybrid thalamic circuits Nature 2002 417 854 858 10.1038/nature00825 12075353

